# Construction and investigation of β3GNT2-associated regulatory network in esophageal carcinoma

**DOI:** 10.1186/s11658-022-00306-y

**Published:** 2022-01-24

**Authors:** Zhiguo Luo, Qing Hu, Yuanhui Tang, Yahui Leng, Tian Tian, Shuangyue Tian, Chengyang Huang, Ao Liu, Xinzhou Deng, Li Shen

**Affiliations:** 1 Department of Clinical Oncology, Taihe Hospital, Hubei University of Medicine, 30 South Renmin Road, Shiyan, 442000 Hubei China; 2 Institute of Basic Medical Sciences, Hubei University of Medicine, Shiyan, China

**Keywords:** Esophageal carcinoma, Progression, Glycosyltransferase, β3GNT2

## Abstract

**Background:**

Glycosyltransferases play a crucial role in various cancers. β1, 3-*N-*acetylglucosaminyltransferase 2, a polylactosamine synthase, is an important member of the glycosyltransferase family. However, the biological function and regulatory mechanism of β3GNT2 in esophageal carcinoma (ESCA) is still poorly understood.

**Methods:**

The Cancer Genome Atlas and Genotype-Tissue Expression databases were used for gene expression and prognosis analysis. Quantitative real-time PCR, Western blot, and immunohistochemistry were performed to detect the expression of β3GNT2 in ESCA cell lines and tissues. In vitro assays and xenograft tumor models were utilized to evaluate the impact of β3GNT2 on ESCA progression. The downstream effectors and upstream regulators of β3GNT2 were predicted by online software and verified by functional experiments.

**Results:**

We found that β3GNT2 was highly expressed in ESCA tissues and positively correlated with poor prognosis in ESCA patients. β3GNT2 expression was closely associated with the tumor size, TNM stage, and overall survival of ESCA patients. Functionally, β3GNT2 promoted ESCA cell growth, migration, and invasion in vitro, as well as tumorigenesis in vivo. Mechanistically, β3GNT2 knockdown decreased the expression of the polylactosamine on EGFR. Knockdown of β3GNT2 also inhibited the JAK/STAT signaling pathway. Meanwhile, the JAK/STAT inhibitor could partly reverse the biological effects caused by β3GNT2 overexpression. Moreover, β3GNT2 expression was positively regulated by CREB1 and negatively regulated by miR-133b. Both CREB1 and miR-133b was involved in the β3GNT2-mediated ESCA progression.

**Conclusions:**

Our study, for the first time, reveals the importance of β3GNT2 in ESCA progression and offers a potential therapeutic target for ESCA.

**Supplementary Information:**

The online version contains supplementary material available at 10.1186/s11658-022-00306-y.

## Background

Esophageal carcinoma (ESCA) is one of the most common cancers around the world [1]. China is a high incidence area for ESCA and approximately 50% of ESCA cases worldwide occur in China [2]. Although great progress has been made in the treatment of ESCA, such as surgery, radiotherapy, and chemotherapy, the five-year survival rate of ESCA patients is still less than 20% [3]. This is mainly due to the lack of effective diagnostic and prognostic biomarkers. Moreover, the mechanism involved in the occurrence and development of ESCA has not been fully elucidated. Thus, it is of great importance to explore the pathogenesis of ESCA and develop effective therapies for ESCA patients.

Glycosylation is a ubiquitous post-translational modification of proteins [4, 5]. Alteration of glycosylation, as a hallmark of cancer, is linked to tumor initiation, progression, and metastasis [6, 7]. Glycosylation is generated by a variety of glycosyltransferases through complex biosynthetic pathways. More than 200 glycosyltransferases have been reported so far [8]. Dysregulated expression of glycosyltransferases has been implicated in ESCA. For example, high expression of C1GALT1 and OGT was associated with lymph node metastasis of ESCA patients [9, 10]. FUT8 was identified as a key driver for radioresistance in ESCA [11]. However, a comprehensive understanding of glycosyltransferases in ESCA is still lacking.

In this study, The Cancer Genome Atlas (TCGA) and Genotype-Tissue Expression (GTEx) databases were used to identify differentially expressed glycosyltransferases between ESCA tissues and normal tissues. We found that β1, 3-*N*-acetylglucosaminyltransferase 2 (β3GNT2) was overexpressed in ESCA tissues and correlated with the poor prognosis of ESCA patients. We further investigated the biological function and regulatory mechanism of β3GNT2 in ESCA progression. Our results suggest that β3GNT2 may serve as a potential therapeutic target for ESCA.

## Materials and methods

### Data acquisition and processing

RNA-seq data, miRNA-seq data, and the corresponding clinical information were obtained from the TCGA (https://tcga-data.nci.nih.gov/tcga/) and GTEx (http://gtexportal.org/home/) platforms. Differentially expressed glycosyltransferases, transcription factors, miRNAs were screened using the DESeq2 R package. An adjusted *P*-value < 0.05 and |log2 fold-change (FC)|> 0.5 were set as the thresholds. Volcano plots and heatmaps were generated using the R package ggplot and pheatmap, respectively. A prognostic nomogram was constructed by R software with the rms package. The Receiver Operating Characteristic (ROC) curves were plotted and the area under the ROC curve was calculated using the R package pROC. Survival prediction was performed using the GEPIA database (http://gepia.cancer-pku.cn/). Gene Set Enrichment Analysis (GSEA) analysis was carried out using the GSEA software (http://www.broadinstitute.org/gsea). Co-expression analysis was determined by using the LinkedOmics platform (http://www.linkedomics.org/).

### Tissue collection

Sixty-five tumor tissues and paired paracancerous tissues were collected from patients who were diagnosed with ESCA and received surgery at the Taihe Hospital, Hubei University of Medicine (Shiyan, China) between 2008 and 2012. The patients, including 35 males and 30 females, were aged 35–75 years with an average age of 54.62 ± 9.14. None of the patients underwent any other treatment before surgery. Samples were stored at − 80 °C until further processing. The studies involving human participants were approved by the Ethics Committee of the Hubei University of Medicine. All patients provided informed written consent.

### Cell culture and transfection

Human ESCA cell lines TE-1, KYSE150, and KYSE410 were purchased from Procell (Wuhan, China). These cells were cultured in RPMI-1640 medium (Gibco, Carlsbad, CA, USA) containing 10% FBS at 37 °C, 5% CO_2_. The pcDNA3.1/β3GNT2 plasmid, pcDNA3.1/CREB1 plasmid, empty pcDNA3.1 plasmid, a lentiviral vector expressing β3GNT2 shRNA, a lentiviral vector expressing CREB1 shRNA, control lentiviral vector, miR-133b inhibitor, inhibitor control, miR-133b mimics, and mimics control were constructed by GenePharma (Shanghai, China). All transfections were conducted with Lipofectamine 3000 reagent (Invitrogen, Carlsbad, CA, USA) according to the manufacturer’s protocol. Stable clones were selected by puromycin or G418.

### Quantitative real-time PCR (qPCR) and Western blot

Total RNA was extracted using RNAiso Plus (Takara, Dalian, China). For mRNA detection, cDNA was synthesized using the PrimeScript RT Reagent Kit (Takara) and qPCR was carried out employing the SYBR-Green PCR master mix (Takara). For miRNA analysis, the One-Step PrimeScript miRNA cDNA synthesis kit (Takara) and the SYBR PrimeScript miRNA RT-PCR Kit (Takara) were used. Data were normalized to GAPDH or U6 based on the 2^−ΔΔCt^ method. All primers were synthesized by GenePharma. Total protein was isolated using RIPA lysis buffer (Beyotime, Shanghai, China). Western blot was conducted as previously described [11]. Primary antibodies were as follows: β3GNT2 (ab236291), CREB1 (ab32515), JAK1 (ab133666), STAT3 (ab68153), p-STAT3 (ab267373), EGFR (ab52894), and GAPDH (ab8245). All antibodies were acquired from Abcam (Shanghai, China).

### Immunohistochemistry (IHC)

Tissues were fixed in formalin and embedded in paraffin. Afterward, 5 µm sections were prepared for IHC staining. PV-9000 two-step immunohistochemical kit (ZSGB-BIO, Beijing China) was used to detect the expression of β3GNT2 protein. The positive signal was visualized by DAB detection Kit (ZSGB-BIO). Slides were read by two pathologists using a Nikon Eclipse E600 microscope (Nikon, Tokyo, Japan). IHC score = intensity score × percentage score [12]. β3GNT2 expression was defined as follows: low expression (score 0–4) and high expression (score ≥ 5).

### Cell proliferation and colony formation assays

Cell proliferation was detected by Cell Counting Kit-8 (CCK-8) assay (Dojindo, Kumamoto, Japan). Cells (1 × 10^3^ per well) were seeded into a 96-well plate. Absorbance was measured at 450 nm. For colony formation assay, cells were added into a 6-well plate at a density of 5 × 10^3^ cells/well. After 2 weeks, colonies (> 50 cells) were stained with 0.5% crystal violet (Beyotime).

### Cell migration and invasion assays

Cells (5 × 10^4^ in 200 μL serum-free medium) were seeded into the upper chamber of the Transwell apparatus (Corning Incorporated, Corning, NY, USA). Transwell chambers precoated with or without Matrigel (BD Biosciences, San Jose, CA, USA) were used to assess cell invasion and migration, respectively. The lower chamber was filled with 600μL of complete culture medium. After incubation for 36 h, migrated or invaded cells were stained with 0.5% crystal violet.

### Cell apoptosis assay

Cell apoptosis was detected by flow cytometry using the Apoptosis Detection Kit (Absin, Shanghai, China). The percentage of apoptotic cells was defined as the sum of early apoptotic cells and late apoptotic cells. All experimental steps were conducted according to kit manual instructions.

### Lectin pull-down assay

Lycopersicon esculentum lectin (LEL) agarose beads (#AL-1113, Vector Labs, Burlingame, CA, USA) were used to analyze polylactosamine chains on glycoproteins. Cell lysates (0.5 mg) were incubated with 30 μL of LEL-conjugated beads to capture the lectin-glycoprotein complexes. Precipitated glycoproteins were eluted and subjected to Western blot analysis with an anti-EGFR antibody. The EGFR in total lysates was used as the internal loading control.

### Luciferase assay

The wild-type or mutant β3GNT2 3′UTR sequences containing the binding sites for miR-133b were cloned into the pmirGLO vector. Besides, the fragments of β3GNT2 promoter with wild-type or mutant CERB1 binding sites were inserted into a pGL3-basic vector. All the above plasmids were constructed by GenePharma. Cells were harvested 48 h after transfection. Luciferase activities were determined using the dual-luciferase reporter assay system (Promega, Madison, WI, USA).

### Chromatin immunoprecipitation (ChIP) assay

The EZ-Magna ChIP™ Kit (Millipore, Billerica, MA, USA) was used for ChIP assay [13]. Detection was performed according to the manufacturer’s protocol. The binding of CREB1 to the β3GNT2 promoter region was evaluated by qPCR.

### Animal experiments

Female BALB/c nude mice, 4–5 weeks old, were provided by the Animal Center of Hubei University of Medicine. Transfected cells (1 × 10^7^) were subcutaneously injected into nude mice. Tumor volume (0.5 × length × width^2^) was measured every week. Four weeks after injection, all mice were sacrificed.

### Statistical analysis

Data are presented as mean ± SD. Results were analyzed with Graphpad Prism (version 7) software (GraphPad, San Diego, CA, USA). Student’s t-test, Chi-square test, and one-way ANOVA were used to determine statistical significance. A correlation was analyzed using the Pearson correlation coefficient test. Overall survival curves were plotted according to the Kaplan–Meier method. A P-value < 0.05 was considered significant.

## Results

### Identification of differentially expressed glycosyltransferases in ESCA

**Fig. 1:**
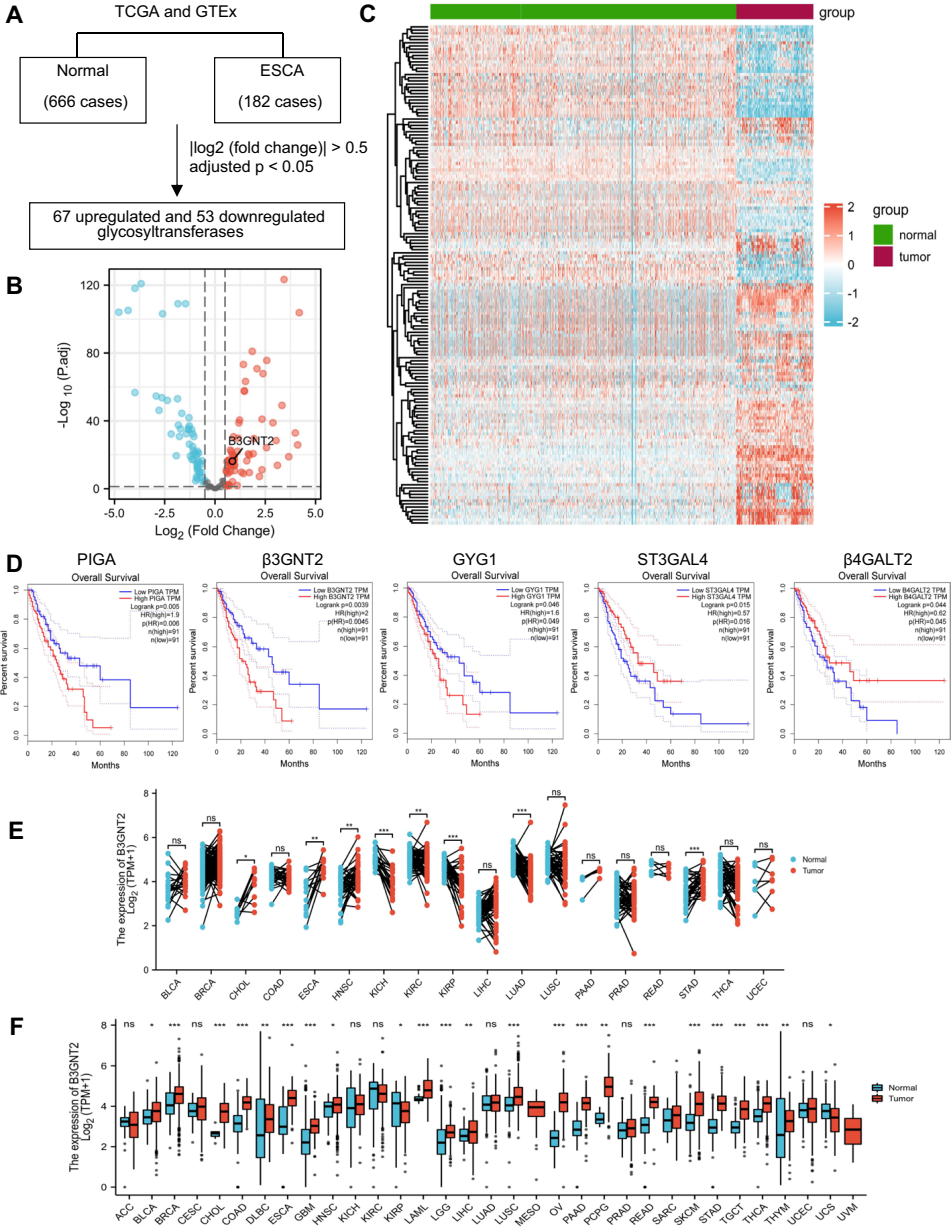
Screening of significantly dysregulated glycosyltransferases in ESCA based on the TCGA and GTEx databases. **A** Flowchart of screening and identification process. **B** Volcano plot showing the distribution of differentially expressed glycosyltransferases. **C** Heatmap showing differential expression patterns of glycosyltransferases. **D** Survival plots for β3GNT2, ST3GAL4, β4GALT2, PIGA, and GYG1. **E** Pan-cancer analysis of β3GNT2 expression in paired tumor tissues and normal tissues. **F** Pan-cancer analysis of β3GNT2 expression in unpaired tumor and normal tissues. Data are expressed as mean ± SD. *NS* no significance; *P < 0.05; **P < 0.01; ***P < 0.001

Using the RNA-seq data from the TCGA and GTEx datasets, we screened differentially expressed glycosyltransferases between ESCA tissues (182 cases) and normal esophageal tissues (666 cases) (Fig. 1A). In total, there were 63 upregulated and 57 downregulated glycosyltransferases in ESCA tissues compared with normal tissues (Fig. 1B, Additional file 1: Table S1). Aberrant expression of glycosyltransferases was presented as a heatmap plot (Fig. 1C). We further investigated the relationship between glycosyltransferases and the overall survival of ESCA patients. Kaplan–Meier analysis showed that only five glycosyltransferases, including β3GNT2, ST3GAL4, β4GALT2, PIGA, and GYG1 were related to overall survival (Fig. 1D, Additional file 1: Table S1). Among them, we observed that β3GNT2 was the most significantly upregulated glycosyltransferase in ESCA (log2|FC|= 0.86591, P < 0.001). Thus, β3GNT2 was expected to be an oncogenic factor. To elucidate the gene expression characteristics of β3GNT2 in human malignancies, pan-cancer analysis was performed using the TCGA and GTEx databases. We found that β3GNT2 expression was increased in most types of cancers (Fig. 1E, F). Based on the above analysis, β3GNT2 was selected for further study.

### Clinical importance of β3GNT2 in ESCA

**Fig. 2:**
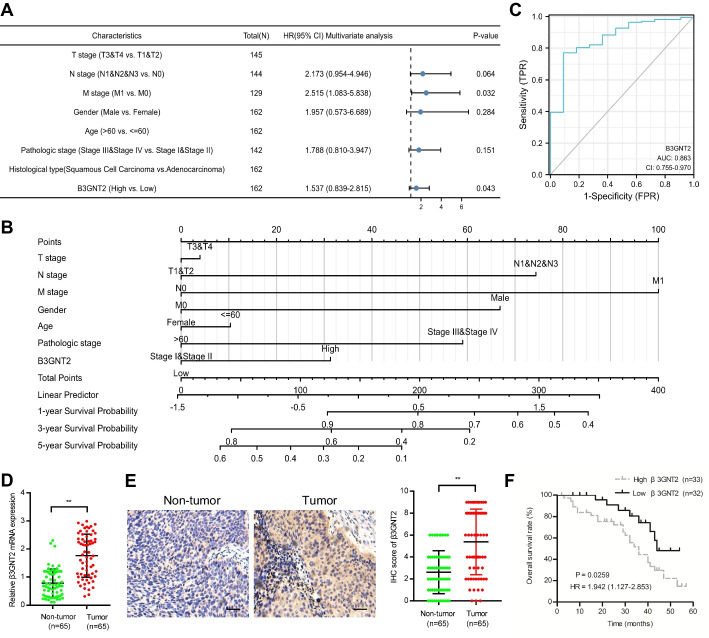
High expression of β3GNT2 in ESCA predicts poor prognosis. **A** Forest plot based on multivariate Cox analysis. **B** A nomogram predicting 1-, 3-, 5-year survival rate. **C** The diagnostic value analysis of β3GNT2 by ROC curve. **D** Analysis of β3GNT2 mRNA expression by qPCR. **E** Analysis of β3GNT2 protein expression by IHC (bar value = 100 μm). **F** Kaplan–Meier analysis of overall survival according to β3GNT2 expression. Data are expressed as mean ± SD. **P < 0.01

To evaluate the prognostic potential of β3GNT2, we downloaded the clinical information of 182 ESCA patients from the TCGA data portal. Analysis was performed using the univariate or multivariate Cox proportional hazards regression (Table 1). The univariate analysis indicated that N stage, M stage, pathologic stage, and β3GNT2 expression could influence overall survival. The multivariate analysis revealed that β3GNT2 was an independent prognostic factor for overall survival (Fig. 2A). Based on the multivariate analysis, a nomogram prognostic evaluation model was successfully constructed, which could predict the probability of 1-, 3- and 5-year overall survival of patients with ESCA (Fig. 2B). The ROC curve showed that β3GNT2 had a relatively high diagnostic accuracy, representing its strong potential for distinguishing normal samples from ESCA samples (Fig. 2C). To validate the findings obtained from public databases, we collected 65 ESCA specimens. The results of qPCR and IHC demonstrated that β3GNT2 was upregulated in ESCA tissues (Fig. 2D, E). According to Kaplan–Meier analysis, high β3GNT2 expression was linked to unsatisfactory survival in patients with ESCA (Fig. 2F). By analyzing the pathological features, we found that β3GNT2 expression was associated with the tumor size and TNM stage (Table 2). Thus, β3GNT2 could be a valuable prognostic marker for ESCA, and upregulation of β3GNT2 might contribute to ESCA development.

**Table 1:** Univariate and multivariate analysis of overall survival (Cox regression model)

Characteristics	Total (N)	Univariate analysis		Multivariate analysis	
		Hazard ratio (95% CI)	P-value	Hazard ratio (95% CI)	P-value
T stage (T3 and T4 vs. T1 and T2)	145	1.312 (0.756–2.277)	0.334		
N stage (N1 and N2 and N3 vs. N0)	144	2.970 (1.606–5.493)	< 0.001	2.173 (0.954–4.946)	0.064
M stage (M1 vs. M0)	129	5.075 (2.312–11.136)	< 0.001	2.515 (1.083–5.838)	0.032
Gender (Male vs. Female)	162	2.306 (0.922–5.770)	0.074	1.957 (0.573–6.689)	0.284
Age (> 60 vs. ≤ 60)	162	0.831 (0.506–1.365)	0.466		
Pathologic stage (III and IV vs. I and II)	142	3.223 (1.807–5.747)	< 0.001	1.788 (0.810–3.947)	0.151
Histological type (Adenocarcinoma vs. squamous cell carcinoma)	162	0.875 (0.526–1.455)	0.607		
β3GNT2 (High vs. low)	162	1.736 (1.041–2.897)	0.035	1.537 (0.839–2.815)	0.043

**Table 2:** Relationship between β3GNT2 expression and clinicopathological features of ESCA patients

Clinicopathological features	Number of patients	β3GNT2 expression		P-value
		Low (n = 32)	High (n = 33)	
Age				
≤ 60	30	16	14	0.418
> 60	35	16	19	
Gender				
Male	35	15	20	0.154
Female	30	17	13	
Lymph node metastasis				
Positive	31	14	17	0.052
Negative	34	18	16	
Tumor size (cm)				
< 5	37	23	14	0.036
≥ 5	28	9	19	
TNM stage				
I + II	35	20	15	0.017
III + IV	30	12	18	

### β3GNT2 is required for ESCA cell growth, migration, and invasion in vitro

**Fig. 3:**
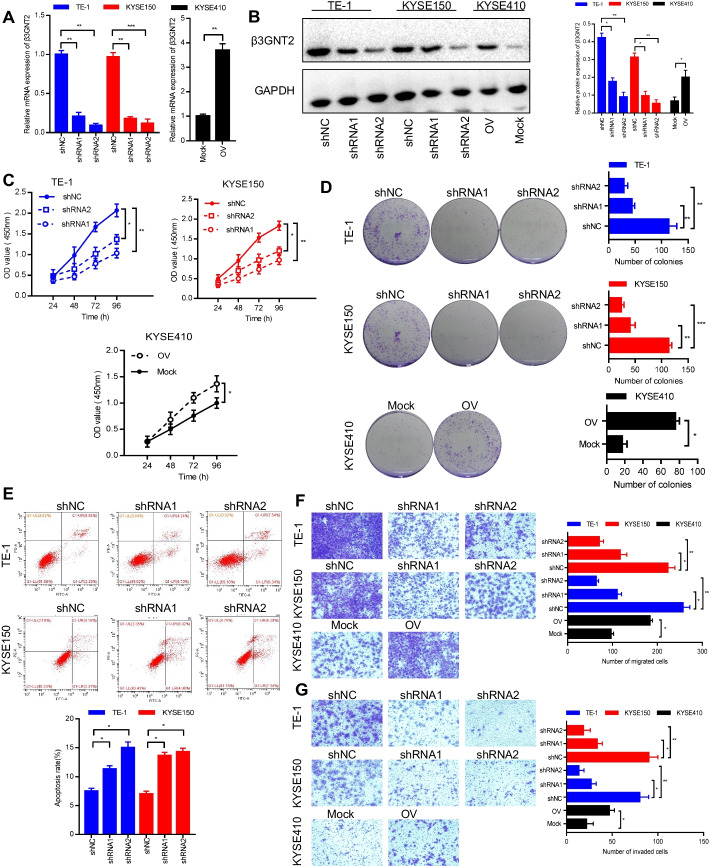
β3GNT2 promotes ESCA cell growth, migration, and invasion in vitro. **A** Analysis of β3GNT2 mRNA expression by qPCR. **B** Analysis of β3GNT2 protein expression by Western blot. **C** Cell proliferation was determined by the CCK-8 assay. **D** Colony formation ability was measured by colony formation assay. **E** Cell apoptosis was assessed by flow cytometry. **F** Cell migratory potential was analyzed by Transwell migration assay. **G** Cell invasion capacity was detected by Matrigel invasion assay. Cells were transfected with the indicated plasmids. shNC, negative control shRNA; shRNAs, β3GNT2 shRNA lentiviral vector; Mock, empty plasmid; OV, pcDNA3.1/β3GNT2 plasmid. Data are expressed as mean ± SD. *P < 0.05; **P < 0.01; ***P < 0.001

To understand the role of β3GNT2 in ESCA, we first analyzed the protein expression of β3GNT2 in three human ESCA cell lines. We found that β3GNT2 expression was highest in TE-1 cells, whereas KYSE150 and KYSE410 cells showed moderate or lower levels of β3GNT2, respectively (Additional file 2: Figure S1). To determine whether β3GNT2 affected cell growth, we conducted loss-of-function studies in TE-1 and KYSE150 cells. The efficiency of β3GNT2 knockdown was verified by qPCR and western blot (Fig. 3A, B). CCK-8 and colony formation assays showed that β3GNT2 knockdown significantly inhibited the proliferation and colony-forming abilities of TE-1 and KYSE150 cells (Fig. 3C, D). To test whether the β3GNT2 knockdown-mediated cell growth inhibition was associated with apoptosis, flow cytometry was carried out. We observed that the proportion of apoptosis in TE-1 and KYSE150 cells was increased following β3GNT2 knockdown (Fig. 3E). Cell migration and invasion are critical steps in tumor metastasis. Transwell migration and Matrigel invasion assays revealed that the migrative and invasive capacities of TE-1 and KYSE150 cells were remarkably decreased after β3GNT2 knockdown (Fig. 3F, G). Accordingly, β3GNT2 shRNA2 was used for subsequent experiments. To validate the effect of β3GNT2 in ESCA progression, we employed the gain-of-function strategy in KYSE410 cells (Fig. 3A, B). Functionally, β3GNT2 overexpression promoted proliferation (Fig. 3C), colony formation (Fig. 3D), migration (Fig. 3F), and invasion (Fig. 3G) of KYSE410 cells. These findings suggested that β3GNT2 exhibited strong oncogenic properties in ESCA.

### β3GNT2 confers in vivo tumorigenicity

**Fig. 4:**
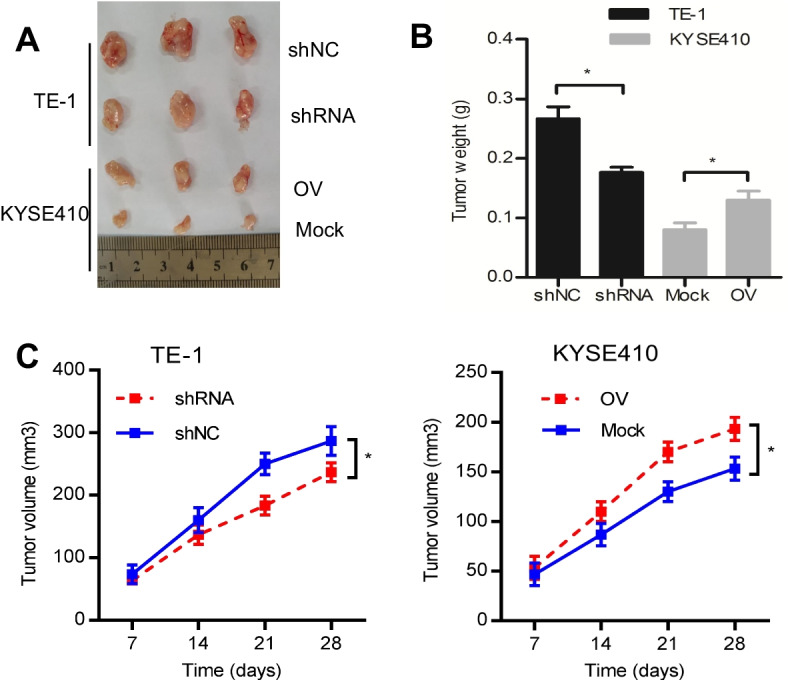
β3GNT2 facilitates ESCA growth in vivo. **A** Representative images of the tumors (n = 5 per group). **B** Measurement of tumor weight in the xenograft model. **C** Quantitative analysis of tumor volume. Cells were transfected with the indicated plasmids. shNC, negative control shRNA; shRNA, β3GNT2 shRNA2 lentiviral vector; Mock, empty plasmid; OV, pcDNA3.1/β3GNT2 plasmid. Data are expressed as mean ± SD. *P < 0.05

Subcutaneous xenograft models were used to explore the function of β3GNT2 in vivo. TE-1 cells with or without the knockdown of β3GNT2 were subcutaneously injected into nude mice. We discovered that β3GNT2 knockdown suppressed tumor growth, as manifested by reduced tumor size (Fig. 4A), tumor weight (Fig. 4B), and tumor volume (Fig. 4C). In contrast, the opposite results were observed in mice injected with KYSE410 cells overexpressing β3GNT2 (Fig. 4A–C). These data implicated that β3GNT2 played an essential role in ESCA tumorigenesis.

### EGFR glycosylation is regulated by β3GNT2

**Fig. 5:**
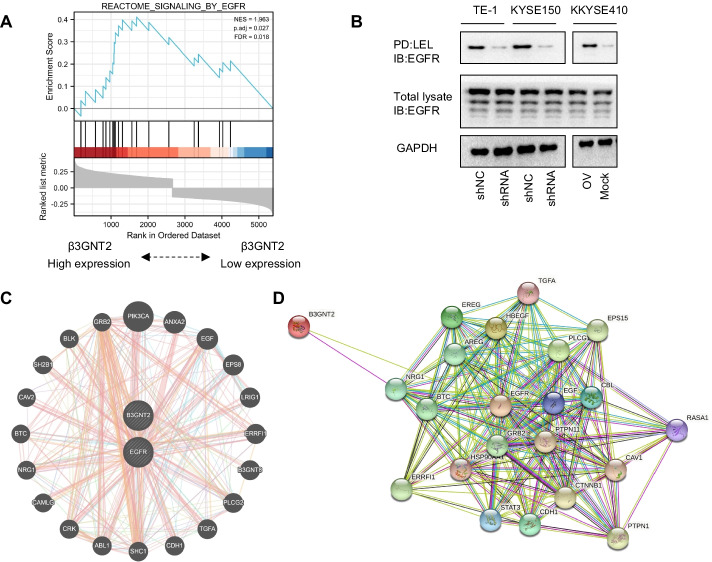
EGFR can be glycosylated by β3GNT2. **A** GSEA plot showing a positive correlation between β3GNT2 expression and EGFR signaling. **B** EGFR pulled down by LEL was analyzed with an anti-EGFR antibody. IB, immunoblot; PD, pull-down. **C** Protein–protein interaction networks were constructed by the online GeneMANIA tool. **D** Protein–protein interaction networks were predicted by the String database. Cells were transfected with the indicated plasmids. shNC, negative control shRNA; shRNA, β3GNT2 shRNA2 lentiviral vector; Mock, empty plasmid; OV, pcDNA3.1/β3GNT2 plasmid

Our pathway analysis from GSEA showed that there was a positive correlation between β3GNT2 expression and EGFR signaling in ESCA (Fig. 5A). β3GNT2 has polylactosamine synthesizing activity and possesses the ability to synthesize polylactosamine structures. To investigate whether EGFR was decorated with polylactosamine and whether C1GALT1 could influence EGFR glycosylation, a lectin pull-down assay was performed. We found that EGFR could be pulled down by LEL and β3GNT2 knockdown reduced the LEL binding to EGFR. In contrast, the overexpression of β3GNT2 had the opposite effects, indicating that polylactosamine chains on EGFR were modulated by β3GNT2 (Fig. 5B). Furthermore, a gene regulatory network was constructed using the GeneMANIA website (http://genemania.org/) and the String database (https://www.string-db.org/) to determine the interactive relationship between β3GNT2 and EGFR (Fig. 5C, D). Hence, EGFR was a major downstream effector of β3GNT2 in ESCA.

### β3GNT2 promotes ESCA progression through the JAK/STAT pathway

**Fig. 6:**
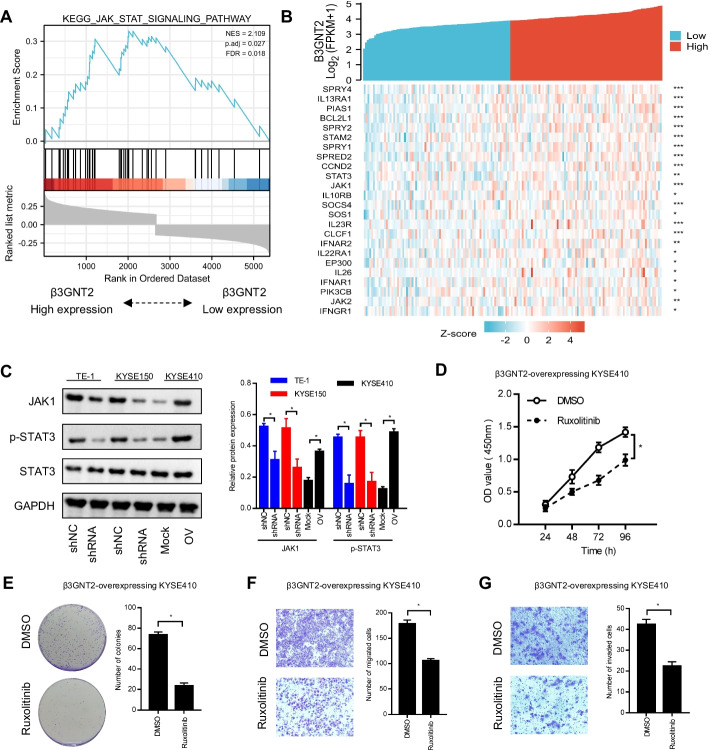
The JAK-STAT pathway is regulated by β3GNT2 in ESCA. **A** GSEA plot showing a positive correlation between β3GNT2 expression and the JAK-STAT pathway. **B** Heatmap showing the Spearman correlation coefficient between β3GNT2 and the JAK-STAT pathway-related genes in ESCA. Asterisks represent the significance of the Spearman correlation. **C** Analysis of the JAK-STAT pathway-related protein expression by Western blot. **D**–**G** The effect of ruxolitinib on cell proliferation, colony formation, migration, and invasion was determined by CCK-8 assay (**D**), colony formation assay (**E**), Transwell migration assay (**F**), and Matrigel invasion assay (**G**). Cells were transfected with the indicated plasmids. shNC, negative control shRNA; shRNA, β3GNT2 shRNA2 lentiviral vector; Mock, empty plasmid; OV, pcDNA3.1/β3GNT2 plasmid. Data are expressed as mean ± SD. *P < 0.05, **P < 0.01, ***P < 0.001

To explore the mechanism underlying the role of β3GNT2 in ESCA progression, genes co-expressed with β3GNT2 in ESCA were analyzed by using the LinkedOmics platform (Additional file 3: Table S2). These genes were then subjected to GSEA analysis. We found that β3GNT2 was closely related to the JAK/STAT pathway (Fig. 6A). The correlation between β3GNT2 and the JAK-STAT pathway-related genes was displayed by the heatmap (Fig. 6B). We discovered that the major proteins of the JAK-STAT pathway such as JAK1 and p-STAT3 were decreased after β3GNT2 knockdown but increased upon β3GNT2 overexpression (Fig. 6C). Then β3GNT2-overexpressing cells were pretreated with the JAK inhibitor, ruxolitinib (1 μM). The CCK-8, colony formation, Transwell migration, and Matrigel invasion assays showed that the promotive effects of β3GNT2 on cell proliferation, colony formation, migration, and invasion were abrogated by ruxolitinib (Fig. 6D–G). These results indicated that the oncogenic potential of β3GNT2 in ESCA was linked to the JAK/STAT pathway.

### CREB1 activates β3GNT2 transcription in ESCA

**Fig. 7:**
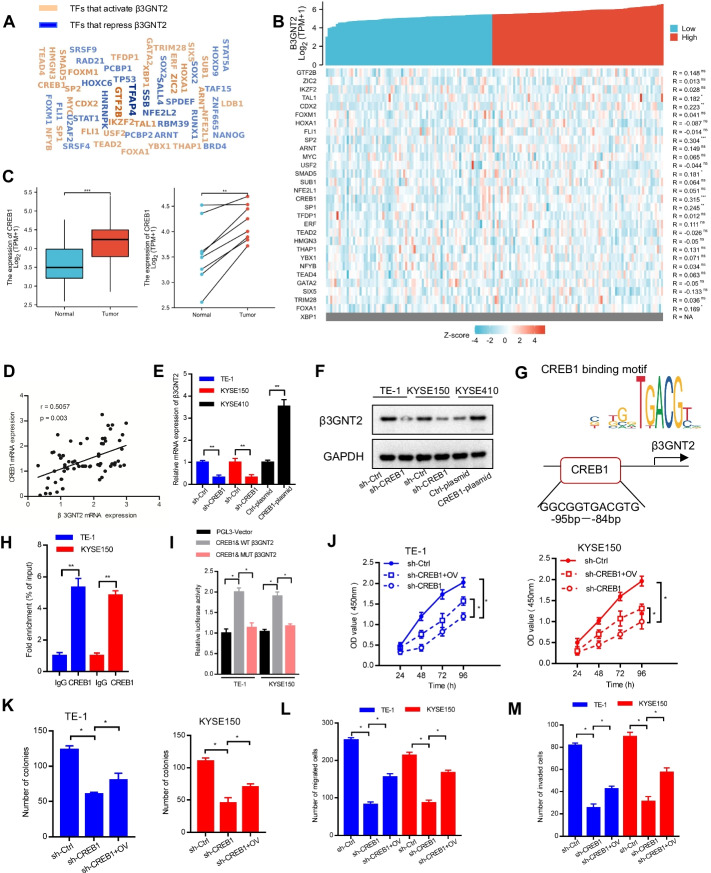
β3GNT2 is a downstream target of CREB1. **A** Transcription factors (TFs) of β3GNT2 predicted by KnockTF. **B** Correlation analysis between transcriptional activators and β3GNT2 based on the TCGA and GTEx databases. **C** Analysis of β3GNT2 expression through the TCGA and GTEx databases. **D** Correlation analysis of CREB1 mRNA expression and β3GNT2 mRNA expression in clinical ESCA samples. **E** Analysis of β3GNT2 mRNA expression by qPCR. **F** Analysis of β3GNT2 protein expression by Western blot. **G** CREB1 binding site in the β3GNT2 promoter region. **H** ChIP-qPCR analysis of CREB1 binding to the β3GNT2 promoter. **I** Dual-luciferase reporter activity assay. **J**–**M** The proliferation, colony formation, migration, and invasion of cells co-transfected with β3GNT2 overexpression plasmid and CREB1 shRNA were measured by CCK-8 assay (**J**), colony formation assay (**K**), Transwell migration assay (**L**), and Matrigel invasion assay (**M**). Cells were transfected with the indicated plasmids. WT, wild-type; MUT, mutant; OV, pcDNA3.1/β3GNT2 plasmid; sh-Ctrl, control shRNA; sh-CREB1, CREB1 shRNA lentiviral vector. Data are expressed as mean ± SD. NA, Data not available; *P < 0.05; **P < 0.01; ***P < 0.001

To clarify the mechanism leading to β3GNT2 upregulation in ESCA, the potential transcription factors involved in the regulation of β3GNT2 were predicted using the online tool KnockTF (http://www.licpathway.net/KnockTF/index.html) (Fig. 7A). The association between transcriptional activators and β3GNT2 in ESCA was analyzed based on the TCGA and GTEx databases (Fig. 7B). Notably, CREB1 was most strongly correlated with β3GNT2 (r = 0.315). Moreover, CREB1 was overexpressed in ESCA (Fig. 7C). A positive correlation was also observed between CREB1 and β3GNT2 in our clinical samples (Fig. 7D). Thus, we speculated that CREB1 could act as an upstream regulator of β3GNT2. To test this hypothesis, we investigated whether β3GNT2 expression was regulated by CREB1. We demonstrated that silencing of CREB1 downregulated β3GNT2 mRNA and protein expression in TE-1 and KYSE150 cells, whereas CREB1 overexpression upregulated the mRNA and protein expression of β3GNT2 in KYSE410 cells (Fig. 7E, F). To identify whether CREB1 could bind to the promoter region of β3GNT2, the JASPAR database (http://jaspar.genereg.net/) was used. Through bioinformatics analysis, one putative CREB1 binding site was identified in the β3GNT2 promoter region (Fig. 7G). The enrichment of CREB1 in the β3GNT2 promoter region was validated by ChIP-qPCR (Fig. 7H). Dual-luciferase reporter assay showed that β3GNT2 transcriptional activity was increased after transfection with CREB1 plasmid. However, mutation of the putative CREB1 binding site attenuated the β3GNT2 promoter activity (Fig. 7I). To explore whether CREB1 participated in the β3GNT2-mediated ESCA progression, we conducted functional rescued experiments. We found that CREB1 silencing inhibited the proliferation, colony formation, migration, and invasion of TE-1 and KYSE150 cells, but this suppression was partially reversed by β3GNT2 overexpression (Fig. 7J–M). In summary, these results suggested that CREB1 transcriptionally upregulated β3GNT2 expression in ESCA.

### miR-133b is another regulatory factor of β3GNT2

**Fig. 8:**
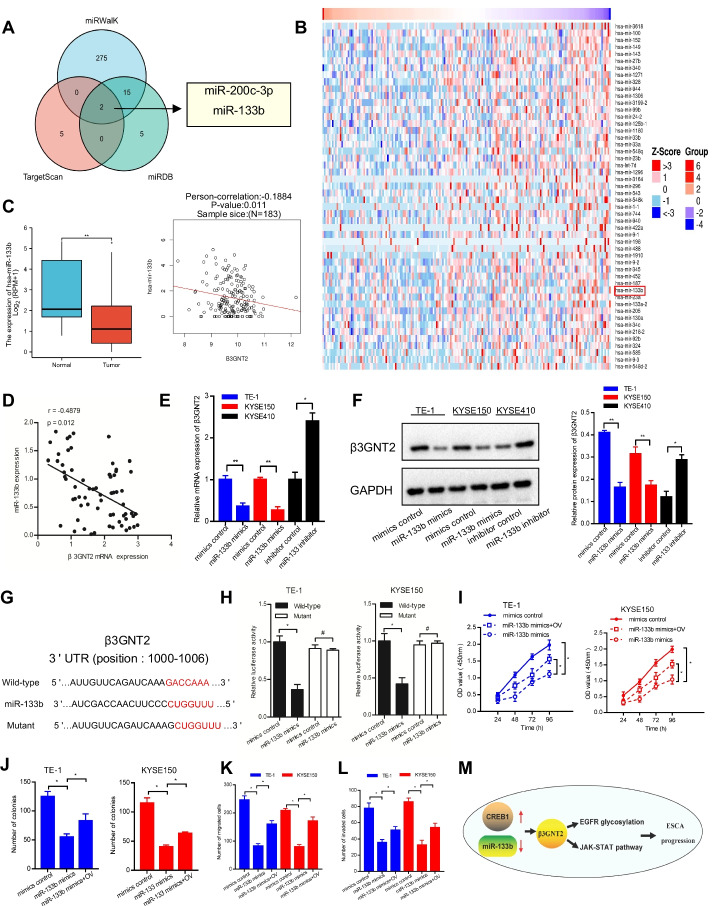
β3GNT2 expression is controlled by miR-133b. **A** Prediction of regulatory miRNAs of β3GNT2. **B** Heatmap showing the top 50 miRNAs negatively correlated with β3GNT2 in ESCA. **C** Analysis of the correlation between miR-133b and β3GNT2 expression based on the TCGA database. **D** Correlation analysis of miR-133b and β3GNT2 expression in clinical ESCA samples. **E** Analysis of β3GNT2 mRNA expression by qPCR. **F** Analysis of β3GNT2 protein expression by Western blot. **G** Putative miR-133b binding site in the β3GNT2 3′-UTR. **H** Dual-luciferase reporter activity assay. **I**–**L** The proliferation, colony formation, migration, and invasion of cells co-transfected with β3GNT2 overexpression plasmid and miR-133b mimics were measured by CCK-8 assay (**I**), colony formation assay (**J**), Transwell migration assay (**K**), and Matrigel invasion assay (**L**). **M** Diagram of the proposed mechanism showing how β3GNT2 regulates ESCA progression. OV, cells transfected with pcDNA3.1/β3GNT2 plasmid. Data are expressed as mean ± SD. ^#^P > 0.05, *P < 0.05; **P < 0.01

Since miRNAs are important post-transcriptional modulators of gene expression, we then investigated whether β3GNT2 was regulated by specific miRNAs [14]. Three publicly available algorithms (TargetScan, miRWalK, and miRDB) were used to predict the potential miRNAs targeting β3GNT2. We found that β3GNT2 was the predicted target of miR-200c-3p and miR-133b (Fig. 8A). In general, miRNA expression is inversely correlated with the expression of target genes. Subsequently, the top 50 negatively related miRNAs for β3GNT2 were identified in the dataset of TCGA-ESCA (Fig. 8B). Remarkably, only miR-133b was downregulated in ESCA, and its expression was negatively associated with β3GNT2 (Fig. 8C). A negative correlation between miR-133b and β3GNT2 was also observed in our clinical samples (Fig. 8D). We further determined whether β3GNT2 expression could be modulated by miR-133b. As expected, miR-133b mimics suppressed β3GNT2 expression both at the mRNA and protein levels in TE-1 and KYSE150 cells, while miR-133b inhibitor enhanced β3GNT2 mRNA and protein expression in KYSE410 cells (Fig. 8E, F). Using the TargetScan algorithm, we discovered that the 3′-UTR of the β3GNT2 gene contained a potential miR-133b binding site (Fig. 8G). Moreover, miR-133b mimics reduced the luciferase activities in TE-1 and KYSE150 cells transfected with wild-type β3GNT2 3′-UTR but not with mutant β3GNT2 3′-UTR (Fig. 8H). Functional experiments demonstrated that the inhibitory effect of miR-133b mimics on cell proliferation, colony formation, migration, and invasion could be restored by β3GNT2 overexpression (Fig. 8I–L). These results suggested that miR-133b targeted β3GNT2 and inhibited its expression in ESCA.

## Discussion

The prognosis of patients with ESCA remains unsatisfactory, despite the use of multiple therapeutic approaches. Further exploration of the diagnostic and prognostic biomarkers for ESCA is warranted. Glycosyltransferases have attracted increasing interest owing to their importance in many biological processes and diseases, including cancer. In the current study, we first found that β3GNT2 expression was upregulated in ESCA. β3GNT2 upregulation was closely associated with the poor prognosis of ESCA patients. Subsequent experiments proved that β3GNT2 contributed to ESCA progression by promoting EGFR glycosylation and activating the JAK-STAT pathway. In addition, CREB1 and miR-133b were critical regulators of β3GNT2 (Fig. 8M). Thus, β3GNT2 may function as an oncogene in ESCA and may be a promising prognostic biomarker for ESCA.

β3GNTs are a family of crucial glycosyltransferases that synthesize a unique glycan structure known as polylactosamine [15]. Abnormal expression of β3GNTs alters the length of polylactosamine chains, which play a critical role in carcinogenesis and cancer progression [16]. β3GNT2, as a member of the β3GNTs, has strong polylactosamine synthesizing activity and is mainly responsible for the elongation of polylactosamine [17]. Dysregulated β3GNT2 expression has been reported in several diseases [18–20]. Our analysis of β3GNT2 expression in clinical samples as well as from public databases both supported that β3GNT2 played an important role in the occurrence and development of ESCA. β3GNT2 knockdown inhibited ESCA growth, migration, and invasion in vitro, as well as tumor formation in vivo. Additionally, ectopic expression of β3GNT2 had the opposite biological function. The present study investigated, for the first time, the oncogenic activity of β3GNT2 in ESCA.

We explored the underlying mechanism of β3GNT2 in promoting ESCA progression. Bioinformatics analysis showed that β3GNT2 expression was linked to the JAK-STAT pathway. Numerous studies revealed that the JAK-STAT pathway was involved in cell proliferation, differentiation, apoptosis, angiogenesis, and immune regulation [21, 22]. The JAK-STAT pathway was also strongly associated with the occurrence of ESCA [23]. In this study, we demonstrated that β3GNT2 promoted ESCA cell proliferation, invasion, and migration via the activation of the JAK-STAT pathway. More importantly, our study further identified EGFR as a downstream effector of β3GNT2 in ESCA. EGFR is a transmembrane glycoprotein and belongs to the tyrosine kinase receptor family [24]. EGFR could be glycosylated with various glycans catalyzed by different glycosyltransferases. For example, OST inhibition disrupted EGFR N-linked glycosylation [25]. Downregulation of GALNT2 suppressed the O-glycosylation of EGFR [26]. The sialylation status of EGFR was related to ST6Gal-I [27]. We confirmed that EGFR carried polylactosamine-type glycans and was a substrate of β3GNT2. These results suggested that β3GNT2 exerted its biological function in ESCA progression mainly through modulating the JAK-STAT pathway and EGFR glycosylation.

To explore the potential factors leading to β3GNT2 upregulation in ESCA, the transcription factors of β3GNT2 were predicted by bioinformatics methods. CREB1 was proposed as a candidate because it had a strong positive correlation with β3GNT2 expression in ESCA. CREB1 is a transcription factor that influences multiple cellular processes, such as cell survival, differentiation, and proliferation [28, 29]. In the present study, we confirmed that CREB1 could bind to the promoter region of β3GNT2 and then enhance its expression. Functional rescued experiments demonstrated that CREB1 silencing suppressed ESCA cell growth, migration, and invasion, but this suppression was reversed by β3GNT2 overexpression. Hence, CREB1 was a key regulator of β3GNT2 in ESCA. To further clarify the upstream regulation mechanism of β3GNT2, the regulatory miRNAs of β3GNT2 were analyzed using the online prediction tools. We discovered that miR-133b could interact with β3GNT2 by binding to its 3′-UTR and there was a negative association between miR-133b and β3GNT2 expression in ESCA. The expression of β3GNT2 was decreased by miR-133b mimics but increased by miR-133b inhibitor. Moreover, the inhibitory effect of miR-133b mimics on ESCA cell growth, migration, and invasion was recovered by β3GNT2 overexpression. It has been reported that miR-133b participates in tumor proliferation and metastasis [30, 31]. Therefore, miR-133b was another critical regulatory factor of β3GNT2 in ESCA.

There are, however, several limitations to our study. First, the expression of polylactosamine chains in ESCA tissues is not examined. Second, the association between the JAK-STAT pathway and EGFR signaling has not been elucidated. The specific glycosylation sites of EGFR remain unknown. The effect of EGFR glycosylation on ESCA progression has not yet been elucidated. Additionally, the mechanism of synergy between CREB1 and miR-133b is not clearly understood. It will be interesting to address these questions in future research.

## Conclusions

Our results confirm that β3GNT2 predicts poor prognosis and acts as an oncogene in ESCA. β3GNT2 can enhance EGFR glycosylation and activate the JAK-STAT pathway. β3GNT2 is transcriptionally upregulated by CREB1. Meanwhile, miR-133b directly targets β3GNT2 and negatively regulates its expression. To our knowledge, this is the first study to explore the biological function, molecular mechanism, and regulatory network of β3GNT2 in ESCA. Thus, β3GNT2 may be a promising prognosis biomarker and a potential therapeutic target for ESCA.

## Supplementary Information

**Additional file 1: Table S1.** Screening of dysregulated glycosyltransferases in ESCA based on the TCGA and GTEx databases.

**Additional file 2: Fig. S1.** Analysis of β3GNT2 protein expression in different ESCA cells by Western blot.

**Additional file 3: Table S2.** Differentially expressed genes correlated with β3GNT2 in ESCA based on the LinkedOmics platform.

## Data Availability

All remaining data are availability within the article and Additional files, or available from the authors upon request.

## Abbreviations

ESCA: Esophageal carcinoma
β3GNT2: β1, 3-*N*-Acetylglucosaminyltransferase 2
TCGA: The Cancer Genome Atlas
GTEx: Genotype-tissue expression
ROC: Receiver operating characteristic
GSEA: Gene set enrichment analysis
LEL: Lycopersicon esculentum lectin
IHC: Immunohistochemistry
qPCR: Quantitative real-time PCR
CCK-8: Cell counting Kit-8
ChIP: Chromatin immunoprecipitation
