# Supplementary figures and images for: Construction and investigation of β3GNT2-associated regulatory network in esophageal carcinoma
Source: Cell Mol Biol Lett. 2022 Jan 24;27:8. doi: 10.1186/s11658-022-00306-y (PMC8903709; doi:10.1186/s11658-022-00306-y)

## Figure S1 Analysis of β3GNT2 protein expression in different ESCA cells by Western blot.

##
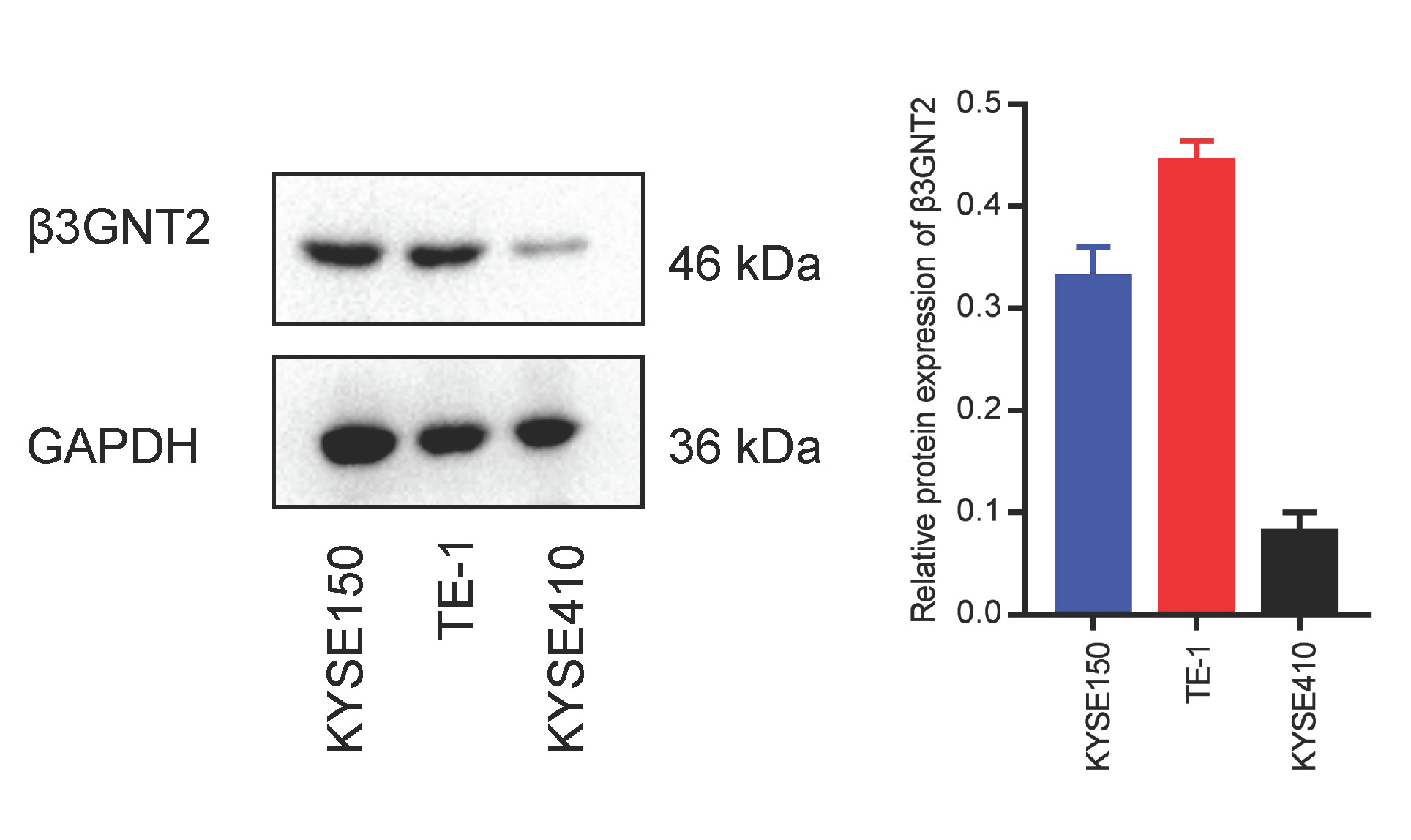

Supplement: Supplementary file 2 — Additional file 2: Fig. S1. Analysis of β3GNT2 protein expression in different ESCA cells by Western blot. [file 11658_2022_306_MOESM2_ESM.doc]
